# Reconstructing Colonization Dynamics of the Human Parasite *Schistosoma mansoni* following Anthropogenic Environmental Changes in Northwest Senegal

**DOI:** 10.1371/journal.pntd.0003998

**Published:** 2015-08-14

**Authors:** Frederik Van den Broeck, Gregory E. Maes, Maarten H. D. Larmuseau, David Rollinson, Ibrahima Sy, Djibril Faye, Filip A. M. Volckaert, Katja Polman, Tine Huyse

**Affiliations:** 1 Department of Biology, University of Leuven, Leuven, Belgium; 2 Department of Biomedical Sciences, Institute of Tropical Medicine, Antwerp, Belgium; 3 College of Marine and Environmental Sciences, James Cook University, Townsville, Australia; 4 Department of Imaging and Pathology, University of Leuven, Leuven, Belgium; 5 Division of Life Sciences, Natural History Museum, London, United Kingdom; 6 UFR Pharmacy, University of Caen Basse-Normandie, Caen, France; 7 Santé Plus, Dakar, Senegal; 8 Department of Biology, Royal Museum for Central Africa, Tervuren, Belgium; René Rachou Research Center, BRAZIL

## Abstract

**Background:**

Anthropogenic environmental changes may lead to ecosystem destabilization and the unintentional colonization of new habitats by parasite populations. A remarkable example is the outbreak of intestinal schistosomiasis in Northwest Senegal following the construction of two dams in the ‘80s. While many studies have investigated the epidemiological, immunological and geographical patterns of *Schistosoma mansoni* infections in this region, little is known about its colonization history.

**Methodology/Principal Findings:**

Parasites were collected at several time points after the disease outbreak and genotyped using a 420 bp fragment of the mitochondrial cytochrome c oxidase subunit 1 gene (*cox*1) and nine nuclear DNA microsatellite markers. Phylogeographic and population genetic analyses revealed the presence of (i) many genetically different haplotypes at the non-recombining mitochondrial marker and (ii) one homogenous *S*. *mansoni* genetic group at the recombining microsatellite markers. These results suggest that the *S*. *mansoni* population in Northwest Senegal was triggered by intraspecific hybridization (i.e. admixture) between parasites that were introduced from different regions. This would comply with the extensive immigration of infected seasonal agricultural workers from neighboring regions in Senegal, Mauritania and Mali. The spatial and temporal stability of the established *S*. *mansoni* population suggests a swift local adaptation of the parasite to the local intermediate snail host *Biomphalaria pfeifferi* at the onset of the epidemic.

**Conclusions/Significance:**

Our results show that *S*. *mansoni* parasites are very successful in colonizing new areas without significant loss of genetic diversity. Maintaining high levels of diversity guarantees the adaptive potential of these parasites to cope with selective pressures such as drug treatment, which might complicate efforts to control the disease.

## Author Summary

*Schistosoma* parasites successfully colonize new regions following the construction of water schemes for power production or agricultural purposes. Here we investigated the colonization history of the human parasite *Schistosoma mansoni* in Northwest Senegal following the construction of two dams in the ‘80s. Parasites were collected at several time points following the disease outbreak and their genetic profile was characterized using molecular markers. Our results showed that many genetically different parasites must have been introduced at the onset of the epidemic, which complies with the extensive immigration of infected seasonal agricultural workers from neighboring regions in Senegal, Mauritania and Mali. Furthermore, we showed that parasite transmission occurred over a large geographic distance, which implies that new alleles, like resistance alleles, could spread rapidly in this system. These new insights demonstrate how colonization following anthropogenic environmental changes may lead to genetically diverse parasite populations within a short time span. High genetic diversity is often linked with a stronger potential to cope with selective pressures such as drug treatment, which may complicate efforts to control the disease.

## Introduction

Environmental change and increasing movements of people, plants and animals have led to species introductions and proliferations into new areas. The colonization, establishment and success of introduced species depend on various biotic and abiotic factors [1–4]. In the case of parasitic organisms, the life cycle is paramount in determining the success of colonization [5]. Parasites with a direct life cycle (when a single host species is involved) can readily invade new areas together with their host [6], while parasites with a complex life cycle need the presence of one or more intermediate host species in order to establish successfully [7,8].

The epidemic outbreak of human intestinal schistosomiasis in Northwest Senegal represents a suitable case study to investigate the evolutionary dynamics of an invasive species. This debilitating disease is caused by the flatworm *Schistosoma mansoni* that per generation cycles through a human final host and a snail intermediate host of the species *Biomphalaria pfeifferi* [9]. As the Senegal River Basin (SRB) suffered from severe droughts during the 1970s and 1980s [10], two dams were built to improve the agricultural conditions for rice production: the Diama dam near the mouth of the Senegal River in 1985 and the Manantali dam upstream in Mali on the Bafing River in 1989 [11]. Subsequent agricultural and hydrological changes were accompanied by 1) strong agro-industrial developments in the village of Richard Toll, resulting in a massive immigration of agricultural workers from neighboring regions in Senegal, Mali and Mauritania [12,13], and 2) major ecological changes such as reduced salinity levels and the formation of open and permanent water bodies and irrigation canals, favoring the growth and spreading of *B*. *pfeifferi* snails [14]. These factors promoted the introduction and successful establishment of *S*. *mansoni* followed by one of the most severe outbreaks of intestinal schistosomiasis ever described [11,13,15–18].

Before the construction of the two dams, human population densities were relatively low in the Delta of the SRB and they were concentrated around Saint-Louis, Ross Béthio and Richard Toll. There were no reports of *S*. *mansoni* and the intermediate snail host *B*. *pfeifferi* was only reported at low densities (< 1% of all collected snails) in the city of Saint-Louis, Lake Guiers and the village Pakh [19]. The first human cases of *Schistosoma mansoni* were reported in 1988 in Richard Toll, the epicenter of the disease outbreak [13]. About 70% of all collected snails were identified as *B*. *pfeifferi* with 44% of them infected with *S*. *mansoni* [20]. The number of cases of intestinal schistosomiasis increased rapidly to epidemic proportions [15,21], and soon after *S*. *mansoni* colonized much of the Delta and part of the Middle Valley of the SRB [18]. Many studies investigated the epidemiological, immunological and geographical patterns of *S*. *mansoni* infections in the SRB, either in single or in mixed infections with *S*. *haematobium* [13,15,16,18,22–29]. A few studies used molecular tools to investigate the distribution of *S*. *mansoni* among hosts [30] and in response to praziquantel drug treatment [31], both on a small geographic scale. However, no study looked at the phylogeography and population genetic structure of *S*. *mansoni* in Senegal at a larger scale and over time.

Here we investigated the evolutionary consequences of the anthropogenic introduction of *S*. *mansoni* in Northwest Senegal since 1988. This is a retrospective study incorporating samples collected at several time points following parasite introduction. In order to understand the dynamics of such a rapid colonization we aimed to reconstruct the epidemic by using nine microsatellite markers and sequences of the mitochondrial cytochrome c oxidase subunit 1 gene (*cox*1). More specifically, we wanted to test whether the current *S*. *mansoni* population was founded by a small number of strains or by multiple introductions from disparate source populations. In the first case, we expected very low levels of genetic diversity and high genetic structure due to genetic drift (e.g. [32,33]). In the second case, we expected average or high levels of genetic diversity, with various potential outcomes of genetic structure depending on the amount of gene flow among the introduced parasite populations (e.g. [34,35]).

## Material & Methods

### Ethics statement

This study is part of a larger investigation on the epidemiology and control of schistosomiasis in Senegal, for which approval was obtained from ‘Le Comité National d'Ethique de la Recherche en Santé’ in Dakar (Senegal), the review board of the Institute of Tropical Medicine Antwerp (Belgium) and the ethical committee of the Antwerp University Hospital (Belgium). Before the start of the study, the respective health authorities, village leaders and school staff of the selected villages were informed about the objectives of the study. These meetings were held in the local language to ensure full comprehension. Informed consent was obtained from teachers and parents or guardians for each participating child. For the village Assoni visited in 2011 in Southwest Senegal, written informed consent was given. For the villages visited in 2006 and 2007 in Northwest Senegal, oral informed consent was given and recorded on paper by assigning ID numbers, name, age, gender and village of residence to those for whom consent was obtained. The data were anonymized prior to analyses. All schistosomiasis positive children were treated with a single dose of praziquantel at 40 mg/kg bodyweight. In schools or classes where the percentage of *Schistosoma* infections was more than 50%, mass treatment of all children was carried out at the end of the study according to WHO guidelines [36].

### Parasite collection and epidemiological background

**Fig 1:**
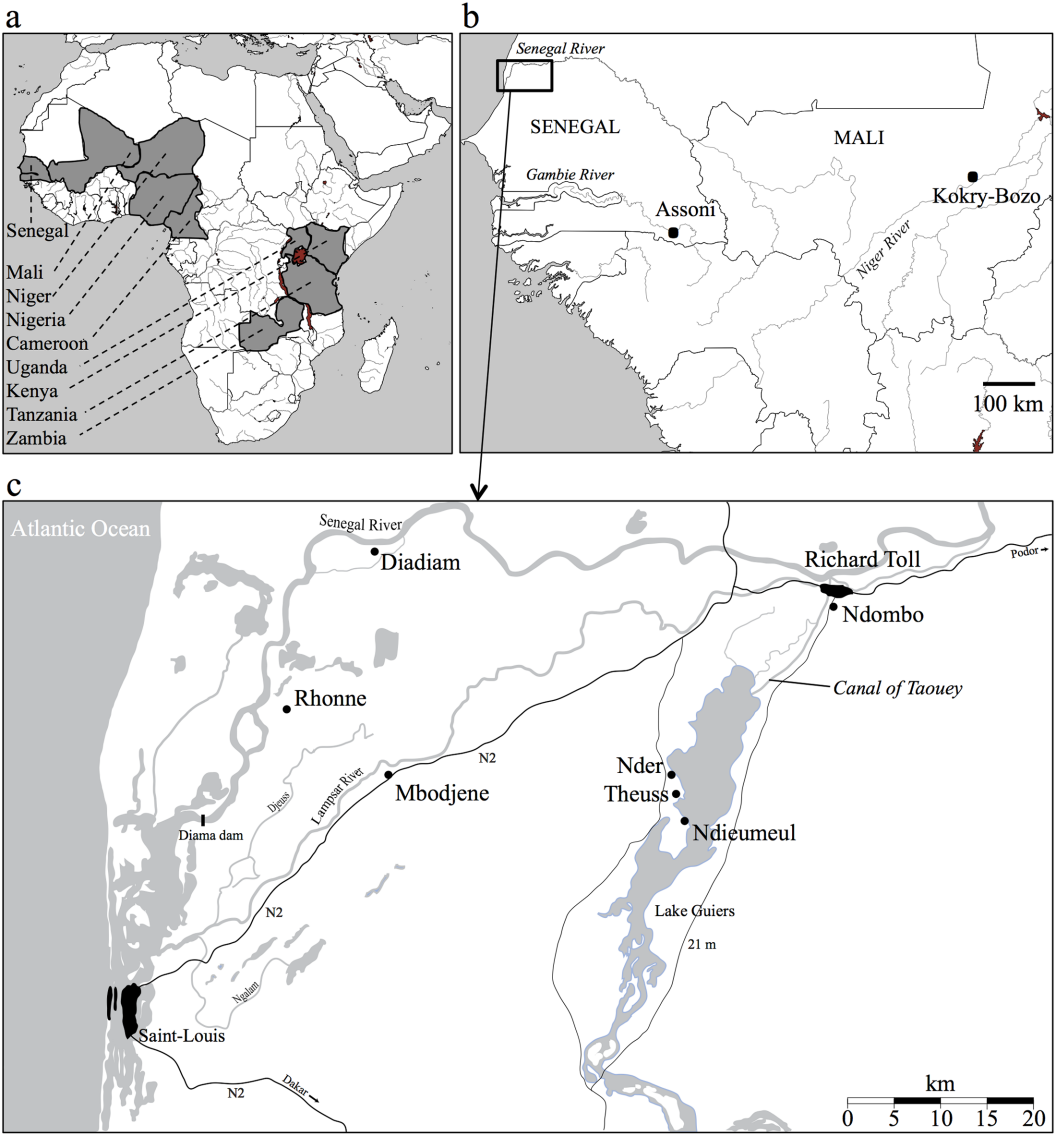
Geographic location of *Schistosoma mansoni* samples. Panel (a) shows the African countries (shaded areas) for which sequence data was generated in this study or obtained from previous studies [38]. Panel (b) and (c) show the location of the villages in Mali and Senegal for which microsatellite data was generated in this study or obtained from previous studies [42]. Panel (c) shows a detailed map of the lower valley of the Senegal River Basin with sampling locations. Detailed information about all samples used in this study are listed in Table 1.

Three genetic datasets were prepared (Table 1). The first genetic dataset, hereafter referred to as DSEQ, comprised the *cox*1 sequences that were generated in this study from the villages Richard Toll (1993 and 1994), Ndombo (1997 and 2006), Assoni (2011) and the villages Wayowayanko and Farako in Southwest Mali (1993) (Table 1) (see section ‘Molecular analyses’ for details on sequencing). Miracidia from the village Assoni were collected within the framework of this study (see below), while all other samples were adult worms provided by the Schistosomiasis Collection at the Natural History Museum in London (SCAN) [37] (SCAN numbers: 2800, 2916, 2953, 3108, 3109, 3421, 3445, 3464, 3465, 3815, 4580). Note that sequences could not be generated for miracidia sampled in 2007 in Northwest Senegal as there was insufficient DNA. Our sequence data were therefore complemented with previously published GenBank sequences from miracidia obtained from Northwest Senegal in the villages Temey and Nder in 2007, from Southeast Senegal in the village Kolda in 2009, and from seven other countries in Africa (Fig 1a and Table 1) [38]. Sequences of cercariae and adults worms from Webster and colleagues [38] were not included here as they might be clones from each other, possibly introducing a bias in estimates of diversity. In contrast, sequences generated from worms in this study were included because the microsatellite genotyping allowed us to identify and exclude clones (see below).

**Table 1:** Details of the three genetic datasets that were used in this study. Collection of microsatellite genotypes and cytochrome *c* oxidase subunit 1 sequences that were obtained from *Schistosoma mansoni* samples during this study or during previous studies [38,42]. The region and year of sampling and the type of sample used as source for DNA template are listed for each sample. Note that the number of samples for each dataset reflects the total number of samples that were successfully genotyped for all loci.

Region	Water course	Village	GPS	Year	Type	DMS1	DMS2	DSEQ	AC DSEQ	Reference
Northwest Senegal	Senegal River	Rhonne	16°20'01"N 16°17'46"W	2007	Miracidia	98	121	/	/	This study
		Diadiam	16°30'24"N 16°12'04"W	2007	Miracidia	6	8	/	/	This study
		Richard Toll	16°28'08"N 15°41'09"W	1993	Worms^SCAN^	7	7	8	KP343660-65	This study
				1994	Worms^SCAN^	12	22	30	KP343666-72	This study
				2007	Miracidia	11	14	/	/	This study
	Canal de Taouey	Ndombo	16°26'23"N 15°41'54"W	1997	Worms^SCAN^	53	62	46	KP343653-59	This study
				2006	Worms^SCAN^	5	7	7	KP343650-52	This study
	Lake Guiers	Temey	16°19'46"N 15°46'04"W	2007	Miracidia	/	/	69	JQ289678-87	[38]
		Theuss	16°14'19"N 15°52'04"W	2006	Miracidia	7	18	/	/	This study
				2007	Miracidia	67	68	/	/	This study
		Nder	16°16'00"N 15°52'28"W	2007	Miracidia	89	152	81	JQ289655-73	[38]
		Ndieumeul	16°13'12"N 15°51'36"W	2007	Miracidia	15	17	/	/	This study
	Lampsar River	Mbodjene	16°13'06"N 16°14'57"W	2007	Miracidia	18	21	/	/	This study
Southeast Senegal	Gambia River	Assoni	12°36'28"N 12°30'07"W	2011	Miracidia	154	168	27	KP343641-46	This study
	Gambia River	Kolda	12°53'22"N 14°56'31"W	2009	Miracidia	/	/	4	JQ289688-90	[38]
Southwest Mali	Niger River	Wayowayanko	12°36'46"N 8°02'50"W	1993	Worms^SCAN^	/	/	2	KP343647-48	This study
	Niger River	Farako	13°24'36"N 6°23'11"W	1993	Worms^SCAN^	/	/	1	KP343649	This study
	Niger River	Kokry-Bozo	13°57'36"N 5°30'36"W	2007	Miracidia	/	73	/	/	[42]
Cameroon	/	Bessoum	/	2007	Miracidia	/	/	11	JQ289588-95	[38]
Coastal Kenya	/	Rekeke	/	2007	Miracidia	/	/	85	JQ289596-617	[38]
Niger	/	Namarigoungou	/	2007	Miracidia	/	/	133	JQ289624-40	[38]
	/	Diambala	/	2007	Miracidia	/	/	27	JQ289643-50	[38]
Nigeria	/	Nebbi	/	2003	Miracidia	/	/	5	JQ28962-3	[38]
Uganda	/	Tonya	/	2007	Miracidia	/	/	16	JQ289711	[38]
	/	Bugoto	/	2009	Miracidia	/	/	5	JQ289712-15	[38]
	/	Walakuba	/	2008	Miracidia	/	/	10	JQ289721-26	[38]
Tanzania	/	Humayebe	/	2008	Miracidia	/	/	44	JQ289691-710	[38]
Zambia	/	Kaunga	/	2008	Miracidia	/	/	40	JQ289727-38	[38]
	/	Siamikobo	/	2008	Miracidia	/	/	6	JQ289739-41	[38]
**Total**						**542**	**758**	**657**		

Worms^SCAN^: worms obtained from the Schistosomiasis Collection at the Natural History Museum in London [37]. DMS1: microsatellite dataset 1. DMS2: microsatellite dataset 2. DSEQ: *cox*1 dataset. AC DSEQ = accession number of *cox*1 sequences from Genbank; only unique sequences (haplotypes) were submitted to GenBank.

The second genetic dataset, hereafter referred to as DMS1, comprised parasites that were genotyped at nine microsatellite markers (see section ‘Molecular analyses’ for details on microsatellite genotyping), and that were either miracidia collected within the framework of this study or adult worms provided by SCAN [37] (see Table 1 for details on sample origin and sample type). These parasites originated from eight villages in Northwest Senegal and from one village in Southeast Senegal. There are three major water bodies in Northwest Senegal: the Senegal River, the Lampsar River and Lake Guiers, each with their tributaries (Fig 1c). The Senegal River and Lake Guiers are connected in Richard Toll through the Canal of Taouey (Fig 1c). The respective villages enrolled in this study along the Senegal River are Rhonne (2007), Diadiam (2007), Richard Toll (1993, 1994, 2007) and Ndombo (1997, 2006). Note that Ndombo lies near the Canal of Taouey within one-kilometer distance from Richard Toll. Hence, samples from Richard Toll and Ndombo combined (1993–2007) are representative for the epicenter of the disease outbreak. Near Lake Guiers, the following villages were enrolled: Theuss (2006, 2007), Nder (2007) and Ndieumeul (2007). Near the Lampsar River only one village was enrolled, namely Mbodjene (2007). To our knowledge, there were treatments in most of the villages around Lake Guiers in March and April 2006. Detailed information on the treatment history for all villages enrolled in this study is however lacking. In each village, about 75 schoolchildren aged 7 to 14 were selected randomly. From each child one stool sample was collected and examined by Kato Katz (2 slides of 25mg) [39]. Eggs from positive stool samples were isolated after filtration, hatched and miracidia were individually pipetted onto Whatman FTA indicator cards in a volume of 3μl of water as described in [40]. Adult worms, provided by SCAN [37], were obtained after one laboratory passage of naturally collected miracidia and/or cercariae (Table 1). As adult worms may be genetically identical (i.e. clones), microsatellite genotypes obtained from worms were visually inspected to identify identical multilocus genotypes. When such identical genotypes were found within a sample, all but one were removed from the dataset. In addition, miracidia were collected within the framework of this study from eight children aged 5–14 in 2011 in the village Assoni in the Region of Kédougou (Southeast Senegal) (Fig 1b). Six of these children were treated eight months prior to this study. The village is situated near tributaries of the Tiokaye River, itself a tributary of the Gambia River. Assoni was enrolled as pilot village in the national control program of Senegal, namely ‘le Programme National de Lutte contre les bilharzioses’ (PNLB) [41]. The prevalence of *S*. *mansoni* infection in Assoni among children aged 6–14 years was initially 100% in 2006 (i.e. before treatment), but decreased to 50% in 2013 after three treatment rounds (2008, 2010, 2012) with praziquantel to those children that were diagnosed positively for *S*. *mansoni* infection [41]. Similar to above, eggs from positive stool samples were isolated after filtration, hatched and miracidia were individually pipetted onto Whatman FTA indicator cards in a volume of 3 μl of water [40].

The third genetic dataset, hereafter referred to as DMS2, includes the same genotypes as DMS1, but complemented with previously published *S*. *mansoni* genotypes from miracidia that were collected in the village Kokry-Bozo in 2007 in Southwest Mali within the framework of the study of Gower and colleagues [42]. Main local watercourses are irrigation channels fed by the Niger River. The prevalence of *S*. *mansoni* infection in Kokry-Bozo at the time of sampling was 88% [42]. Samples were obtained from children that had not been treated previously and miracidia were genotyped at the Department of Infectious Disease Epidemiology (Faculty of Medicine, Imperial College London) [42]. The genetic data of 104 miracidia were provided as raw genotyping chromatogram files that were scored within our lab using GENEMAPPER v4.0 (Applied Biosystems). Six microsatellite markers (*CA11-1*, *S9-1*, *SMD25*, *SMD28*, *SMD89*, *SMDA28*) were shared among the study of Gower and colleagues [42] and our study (see section ‘Molecular analyses’); hence DMS2 only contained six loci. All genotypes were imported into ALLEOGRAM v2.2 [43] for binning of allele lengths. Note that only those samples that were successfully typed at all markers (nine for DMS1 and six for DMS2) were included in the analyses.

### Molecular analyses

Genomic DNA extractions of lab-derived adult worms and naturally collected miracidia were performed with the Nucleospin Tissue kit (Macherey Nagel) following the manufacturer’s instructions. For miracidia, 3 mm discs containing the whole miracidium were excised from the FTA cards and for worms the whole sample was used as DNA source [40].

Sequences of the mitochondrial *cox*1 gene (450 bp) were obtained using primers Asmit-1 and Schisto-3' [44,45] in 25μl PCR reactions, each containing 2 μl of DNA template, 0.5 units of Platinum Taq DNA polymerase (Life Technologies), 1x reaction buffer (Life Technologies), 2 mM MgCl_2_, 0.2 mM dNTPs and 0.8 μM of each primer. PCR conditions were the following: denaturation for 3 min at 95°C, followed by 35 cycles of 45 s at 94°C, 45 s at 49°C, 45 s at 72°C with a final extension of 10 min at 72°C. PCR products were visualized on a 1% agarose gel to check for amplicons, and subsequently purified and sequenced using a Big Dye Chemistry Cycle Sequencing Kit v1.1 in a 3130 Genetic Analyser (Applied Biosystems). The forward primer Asmit-1 was used and complemented with sequencing reverse primer Schisto-3' when sequence quality was poor.

All individual *S*. *mansoni* parasites (both naturally obtained miracidia and lab-derived worms) were genotyped using nine microsatellite loci in a single multiplex reaction (*L46951*, *SMD11*, *S9-1*, *CA11-1*, *SMD25*, *SMD28*, *SMD43*, *SMD89*, *SMDA28* [46–48]) as described in [40]. All PCR products were analyzed using an ABI 3130 Genetic Analyser (Applied Biosystems) and GeneScan 500 LIZ as Size Standard. Allele sizes were manually verified using GENEMAPPER v4.0 (Applied Biosystems).

### Phylogeographic analyses using partial *cox*1 sequences (DSEQ)

All *cox*1 sequences were manually edited and aligned using MUSCLE [49] as implemented in Geneious R6 (http://www.geneious.com/). Species identity was confirmed using BLAST (http://blast.ncbi.nlm.nih.gov/).

Genetic diversity at the *cox*1 fragment (DSEQ) was quantified per region, per village and per year in DNA-SP v5.10.1 [50] by estimating the number of haplotypes (i.e. unique sequences), the haplotype diversity *h* [51], the number of polymorphic sites and the average number of nucleotide differences per site between two randomly chosen DNA sequences (i.e. nucleotide diversity *Π*). Based on the commonly used mutation rate of 10^−8^ mutations per site per year for mitochondrial DNA [52], we assume that the time frame (~ 30 years) is too short to generate new mtDNA lineages, and that mutation will therefore not have affected mtDNA diversity.

Genealogical relationships between all sequences were explored by constructing two haplotype networks based on statistical parsimony [53] in the package ‘pegas’ [54] as implemented in the R software [55]. Haplotypes were first identified using the function *haplotype* and used to construct a network with the function *haploNet*. The number of sequences that represented a given haplotype was logarithmically transformed to narrow high and small values, and used to determine the size of its corresponding pie diagram. A first network included all sequences from the DSEQ dataset. A second network included only sequences from Senegal and Mali.

### Population genetic analyses using microsatellite markers (DMS1 and DMS2)

First, the genetic diversity of parasite populations was quantified by estimating the proportion of heterozygous individuals (i.e. observed heterozygosity, Ho), the expected proportion of heterozygous individuals assuming Hardy-Weinberg Equilibrium (unbiased expected heterozygosity, Hs) and the number of alleles corrected for sample size (allelic richness, AR). Ho and Hs were estimated in GENETIX v4.05 [56] while AR was estimated in the R package ‘hierfstat’ [57]. The inbreeding coefficient *F*_IS_ was estimated by *f* [58] in GENETIX; the significance of *f* was tested using 10,000 permutations and corrected for multiple testing using Bonferroni corrections. Analyses were done per village, year and region.

Second, the ancestry of individual parasites was inferred using a Bayesian Markov Chain Monte Carlo (MCMC) clustering analysis as implemented in STRUCTURE v2.2.3 [59]. The program assigns individuals to *K* populations that are each characterized by a set of allele frequencies. Individuals could be assigned to two or more populations if their genotypes indicate that they are admixed. As *K* is unknown, the model is run multiple times, each time with a different *K-*value (from 1–10). Sampling locations (i.e. village) were included in the model as a prior (LOCPRIOR = 1), as they can assist clustering when the amount of genetic markers is low [60]. Note that the LOCPRIOR model will not falsely identify genetic structure when there is none and will ignore sampling information when the ancestry of individuals is uncorrelated with sampling locations [60]. Five replicate runs were initiated assuming the admixture model and correlated allele frequencies for datasets DMS1 and DMS2; each run consisted of 10^6^ MCMC chains, initiated by 10^5^ burn-in steps. All jobs were run parallel on multiple cores using the R package ‘ParallelStructure’ [61]. The optimal *K* value was identified by the maximum LnP(D), which is the log likelihood of the observed genotype distribution in *K* clusters.

Third, population genetic structure was visualized by exploring the distribution of genotypes of DMS1 and DMS2 in hyperspace using a Factorial Correspondence Analysis (FCA) as implemented in GENETIX and results were visualized using the R software. FCA visualizes genotypic (dis)-similarities among individual parasites.

Fourth, genotypic (dis)-similarities were studied among groups of parasites by estimating the *F*_ST_ analogue *θ* [58]. This was done pairwise between regions and villages in GENETIX for DMS1 and DMS2. Significant population differentiation was tested for all estimates by 1,000 permutations of individuals among localities, and Bonferroni correction was applied for multiple testing. Pairwise estimates of *θ* between villages were visualized with classical multidimensional scaling (CMDS) plots using the R software. Only populations with at least 10 genotypes were kept in order to minimize bias due to low sample size.

## Results

### Characteristics of the datasets

The DSEQ dataset comprised a total of 657 *cox*1 sequences of which 121 were generated in this study (Table 1). After alignment and trimming, sequence fragments of 420 bp long were retained for further analyses.

The DMS1 dataset comprised a total of 542 *S*. *mansoni* parasites that were successfully genotyped for all nine microsatellite loci, among which 154 originated from the village Assoni in Southeast Senegal and 388 from several villages across Northwest Senegal (Table 1). Sample sizes for Northwest Senegal ranged from five genotypes in Ndombo in 2006 to 98 genotypes in Rhonne in 2007 (Table 1).

The DMS2 dataset comprised a total of 758 *S*. *mansoni* parasites that were successfully genotyped for all six microsatellite loci. A total of 73 out of 104 genotypes were successfully scored for the Kokry-Bozo sample from Southwest Mali. Sample sizes for Northwest Senegal ranged from seven in Diadiam and Ndombo (2006) to 152 in Nder (Table 1).

### Phylogeographic analyses

Twenty unique *cox*1 haplotypes were found in Northwest Senegal (Table 2), which is currently about one fifth of the total number of haplotypes observed so far in Africa (i.e. 103). Almost all of the haplotypes found in Northwest Senegal were also found within the village Nder (i.e. 19 out of 20). A total of six haplotypes were identified within a single village (Assoni) in Southeast Senegal, of which three haplotypes were unique to this village; the other three haplotypes were shared with Northwest Senegal. In Kolda in Southeast Senegal, three *cox*1 haplotypes were identified of which one was unique to that village, while the other two haplotypes were also found in Northwest Senegal. The three sequences from Wayowayanko and Farako (Southwest Mali) differed from each other but were in common with Northwest Senegal, of which one was also found in Assoni (Southeast Senegal). Haplotype diversity of all parasites found in Northwest Senegal (*h* = 0.847; N = 241) was high compared to other regions in Africa, ranging from 0.543 in Niger (N = 160) to 0.927 in Tanzania (N = 44). Haplotype diversity was 1.000 in Mali, but the sample size was very low (N = 3). In Richard Toll, haplotype diversity in 1993 (N = 8) and 1994 (N = 30) was equal to 0.929 and 0.772 respectively, comparable to other regions in Africa (Table 2). Similarly, nucleotide diversity of all parasites sampled in Northwest Senegal (*Π* = 0.0081) was similar to other populations in Africa. Only parasites sampled in Zambia and Coastal Kenya showed higher levels of nucleotide diversity (Table 2).

**Table 2:** *Schistosoma mansoni* genetic diversity as estimated at a partial fragment of the cytochrome c oxidase subunit 1 gene. Genetic diversity was estimated for samples obtained from Senegal and eight other African countries (see Table 1 for details on data collection). Sequences that were sampled in other countries than Senegal were pooled per country.

Region	Village(s)	Year(s)	N_seq_	N_hap_	N_pol_	*h* (SD)	*Π* (SD)
Northwest Senegal			241	20	23	0.847 (0.012)	0.0081 (0.0001)
	Richard Toll	1993	8	6	9	0.929 (0.084)	0.0079 (0.0017)
		1994	30	7	10	0.772 (0.003)	0.0060 (0.0009)
	Ndombo	1997	46	7	12	0.563 (0.007)	0.0054 (0.0010)
		2006	7	3	4	0.667 (0.160)	0.0032 (0.0014)
	Nder	2007	81	19	22	0.906 (0.014)	0.0087 (0.0005)
	Temey	2007	69	10	14	0.679 (0.059)	0.0078 (0.0007)
Southeast Senegal			31	8	12	0.705 (0.060)	0.0045 (0.0012)
	Assoni	2011	27	6	7	0.638 (0.068)	0.0025 (0.0007)
	Kolda	2009	4	3	9	0.833 (0.222)	0.0127 (0.0034)
Southwest Mali	Wayowayanko-Farako	1993	3	3	4	1.000 (0.074)	0.0064 (0.0024)
Cameroon	Bessoum	2007	11	7	9	0.873 (0.089)	0.0074 (0.0010)
Coastal Kenya	Rekeke	2007	85	18	32	0.860 (0.029)	0.0234 (0.0008)
Nigeria	Nebbi	2003	5	2	6	0.600 (0.175)	0.0086 (0.0025)
Niger	Namarigoungou-Diambala	2007	160	18	28	0.543 (0.047)	0.0056 (0.0009)
Uganda	Bugoto-Walakuba	2008–2009	31	10	19	0.716 (0.080)	0.0052 (0.0012)
Tanzania	Humayebe	2008	44	20	24	0.927 (0.021)	0.0073 (0.0009)
Zambia	Kaunga-Siamikobo	2008	46	14	44	0.884 (0.025)	0.0321 (0.0043)

N_seq_: number of sequences. N_hap_: number of unique haplotypes. N_pol_: number of polymorphic sites. *h*: haplotype diversity. *Π*: nucleotide diversity. SD: standard deviation.

**Fig 2:**
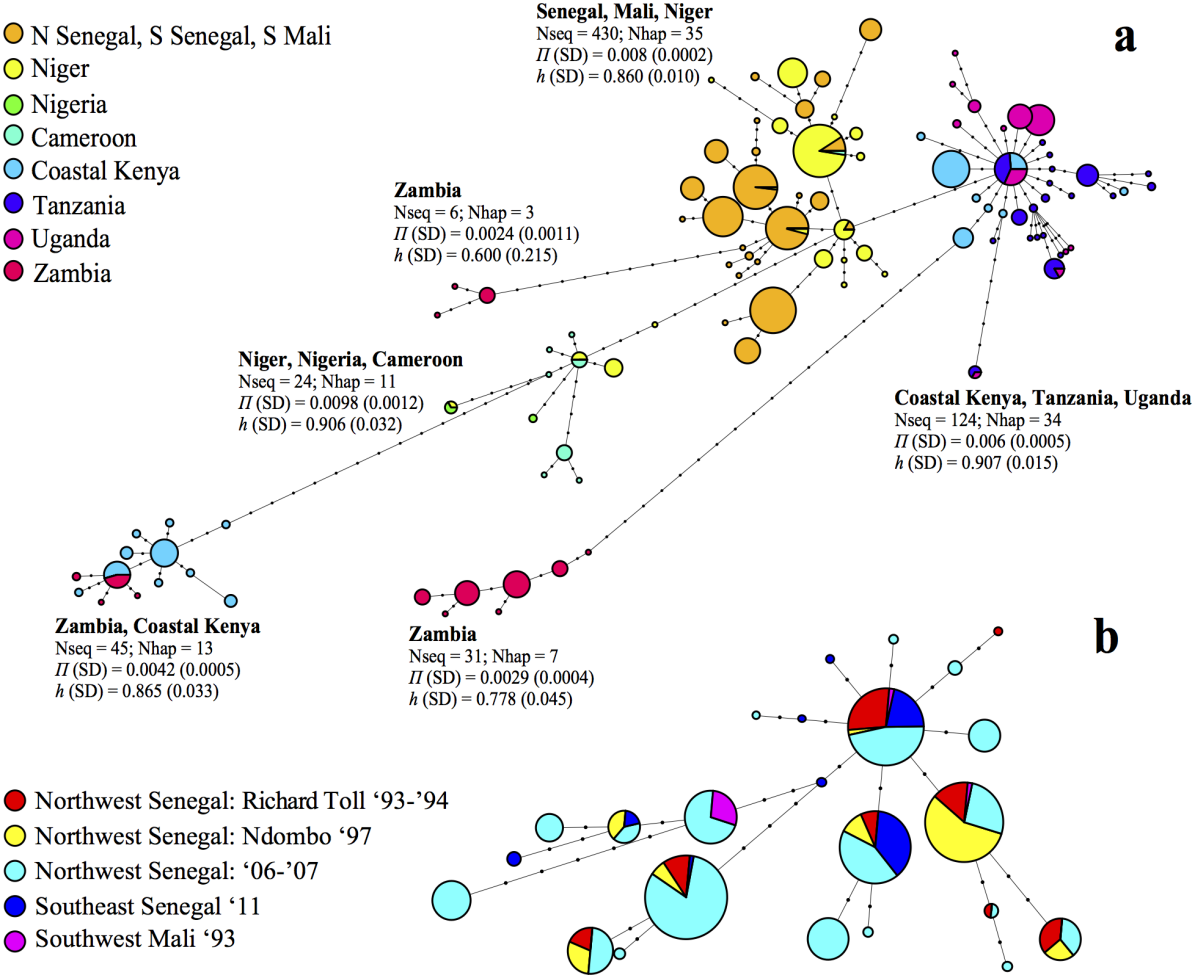
Haplotype networks based on statistical parsimony using partial cytochrome *c* oxidase subunit 1 sequences. The network above (a) comprises all sequences from nine African countries obtained during this study or a previous study [38]. For each phylogeographic group, the number of sequences (Nseq), the number of haplotypes (Nhap), the nucleotide diversity (*Π*) and the haplotype diversity (*h*) with standard deviations (SD) are given. The network below (b) comprises sequences obtained from different villages in Northwest Senegal (1993–2007), Southeast Senegal (2011) and Southwest Mali (2007). Each pie diagram represents a haplotype (i.e. unique sequence) and dots represent haplotypes that were either not sampled or went extinct and can thus be regarded as mutational steps. The sizes of the pie diagrams are in relation to the log transformed number of sequences that represent the respective haplotypes, and the colors indicate the location or year of sampling.

The statistical parsimony network showed that sequences from Northwest Senegal clustered together with haplotypes found in Southeast Senegal, Southwest Mali and some from Niger, and the corresponding haplotype diversity was 0.860 (SD = 0.010) (Fig 2a). Parasites from other regions in Africa were grouped into divergent phylogeographic clades, which were separated from the ‘West-African’ clade by many unsampled or extinct haplotypes (Fig 2a). Haplotype diversity was highest in the ‘East-African’ phylogeographic clade (*h* = 0.907; SD = 0.015) containing parasites from Coastal Kenya, Tanzania and Uganda (Fig 2a). The second network revealed the diversity found in Northwest Senegal, showing many divergent haplotypes that did not cluster according to village or year of sampling (Fig 2b).

### Population genetic analyses

Parasite population genetic diversity as estimated by unbiased expected heterozygosity (Hs), observed heterozygosity (Ho) and allelic richness (AR) was rather uniform across all villages sampled in Northwest Senegal (Table 3), with the exception of Ndombo (2006) that showed lower values of genetic diversity. The diversity levels of *S*. *mansoni* in Northwest Senegal (Hs = 0.38; Ho = 0.36; AR = 2.90) were similar to the diversity in Assoni in Southeast Senegal (Hs = 0.35; Ho = 0.32; AR = 2.85), but slightly lower compared to Kokry-Bozo in Southwest Mali (Hs = 0.45; Ho = 0.42; AR = 3.31) (Table 3).

**Table 3:** *Schistosoma mansoni* genetic diversity estimated from microsatellite markers. Genetic diversity was estimated per village, per region and per year for samples typed at nine (DMS1–542 samples in total) or six (DMS2–758 samples in total) microsatellites markers (see Table 1 for details on data collection).

			DMS1					DMS2				
Region	Village	Year	N_*μ*sat_	Hs	Ho	AR^#^	*F*_IS_	N_*μ*sat_	Hs	Ho	AR^##^	*F*_IS_
Northwest Senegal			388	0.54	0.52	3.74	0.04**	517	0.38	0.36	2.90	0.05**
	Rhonne	2007	98	0.55	0.50	3.74	0.06**	121	0.38	0.35	2.90	0.07*
	Diadiam	2007	6	0.55	0.52	3.73	0.06	8	0.42	0.44	2.93	-0.05
	Richard Toll	1993	7	0.54	0.51	3.83	0.07	7	0.37	0.38	2.83	-0.03
		1994	12	0.55	0.56	3.71	0.03	22	0.38	0.42	2.94	-0.11*
		2007	11	0.57	0.60	3.74	0.06	14	0.42	0.42	2.96	-0.001
	Ndombo	1997	53	0.49	0.48	3.41	0.03	62	0.35	0.33	2.72	0.05
		2006	5	0.46	0.49	3.00	-0.06	7	0.33	0.33	2.17	-0.01
	Theuss	2006	7	0.52	0.44	3.63	0.16**	18	0.37	0.36	2.68	0.01
		2007	67	0.54	0.52	3.72	0.04*	68	0.39	0.38	2.94	0.03
	Nder	2007	89	0.54	0.53	3.71	0.01	152	0.38	0.36	2.85	0.06*
	Ndieumeul	2007	15	0.54	0.47	3.77	0.12*	17	0.37	0.33	2.94	0.11*
	Mbodjene	2007	18	0.54	0.60	3.43	-0.11*	21	0.39	0.44	2.72	-0.13*
Southeast Senegal	Assoni	2011	154	0.50	0.45	3.55	0.12**	168	0.35	0.32	2.85	0.09**
Southwest Mali	Kokry-Bozo	2007	/	/	/	/	/	73	0.45	0.42	3.31	0.06*

DMS1: microsatellite dataset 1. DMS2: microsatellite dataset 2. N_μsat_: number of successfully genotyped parasites. Hs: unbiased expected heterozygosity. Ho: observed heterozygosity. AR: Allelic richness.

^#^: minimum of 10 alleles used for rarefaction.

^##^: minimum of 14 alleles used for rarification.

Statistical significant *F*_IS_ values are given with * for the nominal level of 0.05 and with ** for the nominal level of 0.001.

**Fig 3:**
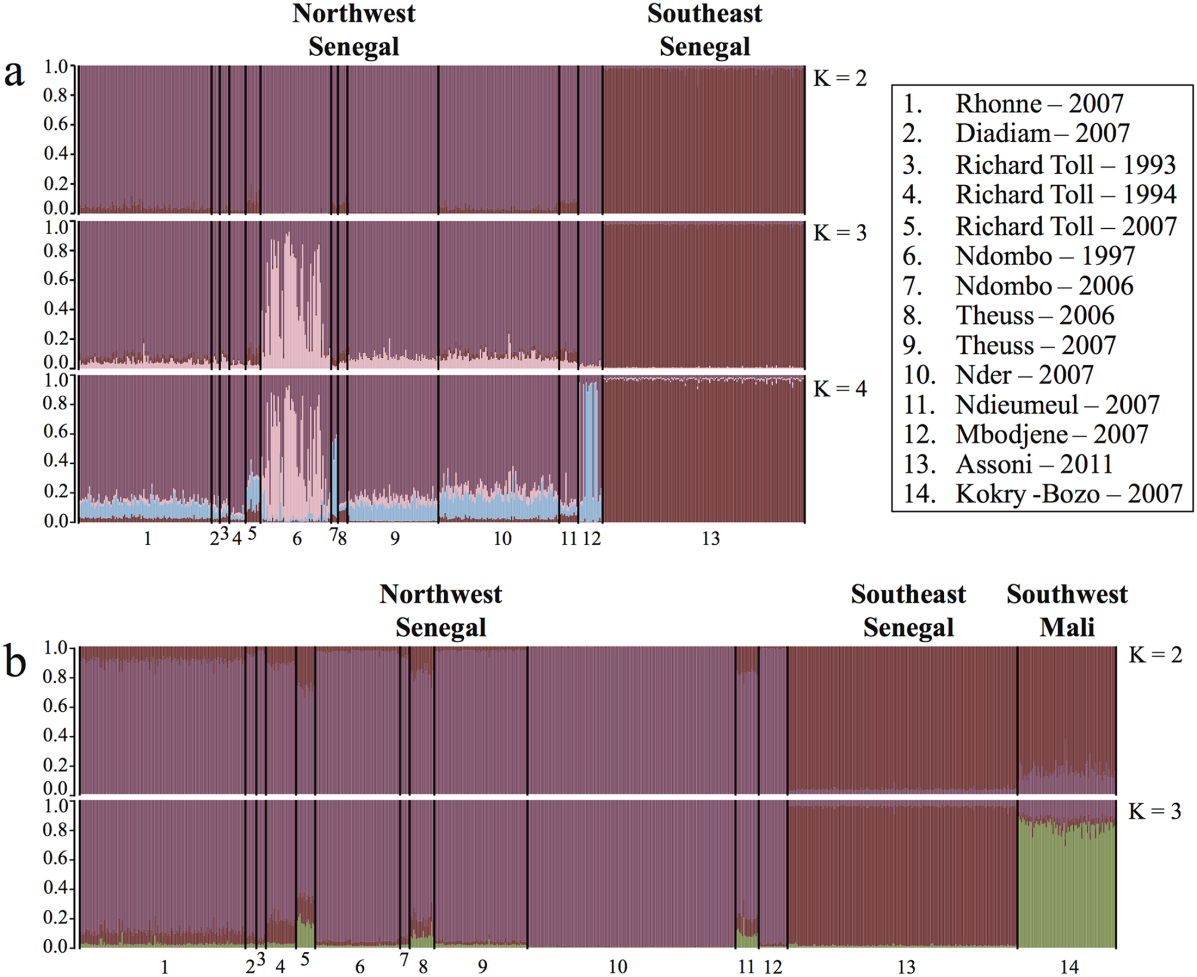
Bayesian clustering analysis with STRUCTURE using microsatellite markers. Each barplot shows the probability on the y-axis (0.0–1.0) of an individual parasite being assigned to a given number of clusters *K* (*K* = 2, 3 or 4) for microsatellite dataset DMS1 (a) and DMS2 (b). Individual parasites are aligned along the x-axis, and grouped according to the location and year of sampling (1–14). Parasites are assigned either to one cluster (each cluster is represented by a different color) or to multiple clusters if their genotypes were admixed (indicated by multiple colors). The optimal *K—*value (*K* = 4 for DMS1 and *K* = 3 for DMS2) was determined by the maximum LnP(D), which is the log likelihood of the observed genotype distribution in *K* clusters.

The highest log likelihood values as estimated in STRUCTURE were found for *K* = 4 for DMS1 and for *K* = 3 for DMS2; the log likelihood decreased thereafter for larger values of *K*. For DMS1, parasites from almost all villages sampled in Northwest Senegal were assigned to one genetic cluster, with the exception of parasites from Ndombo (1997) and Mbodjene (2007) (Fig 3a). In addition, parasites from Assoni were assigned to a separate genetic cluster (Fig 3a). For DMS2, three genetic clusters were identified that were concordant with the three regions Northwest Senegal, Southeast Senegal and Southwest Mali (Fig 3b).

**Fig 4:**
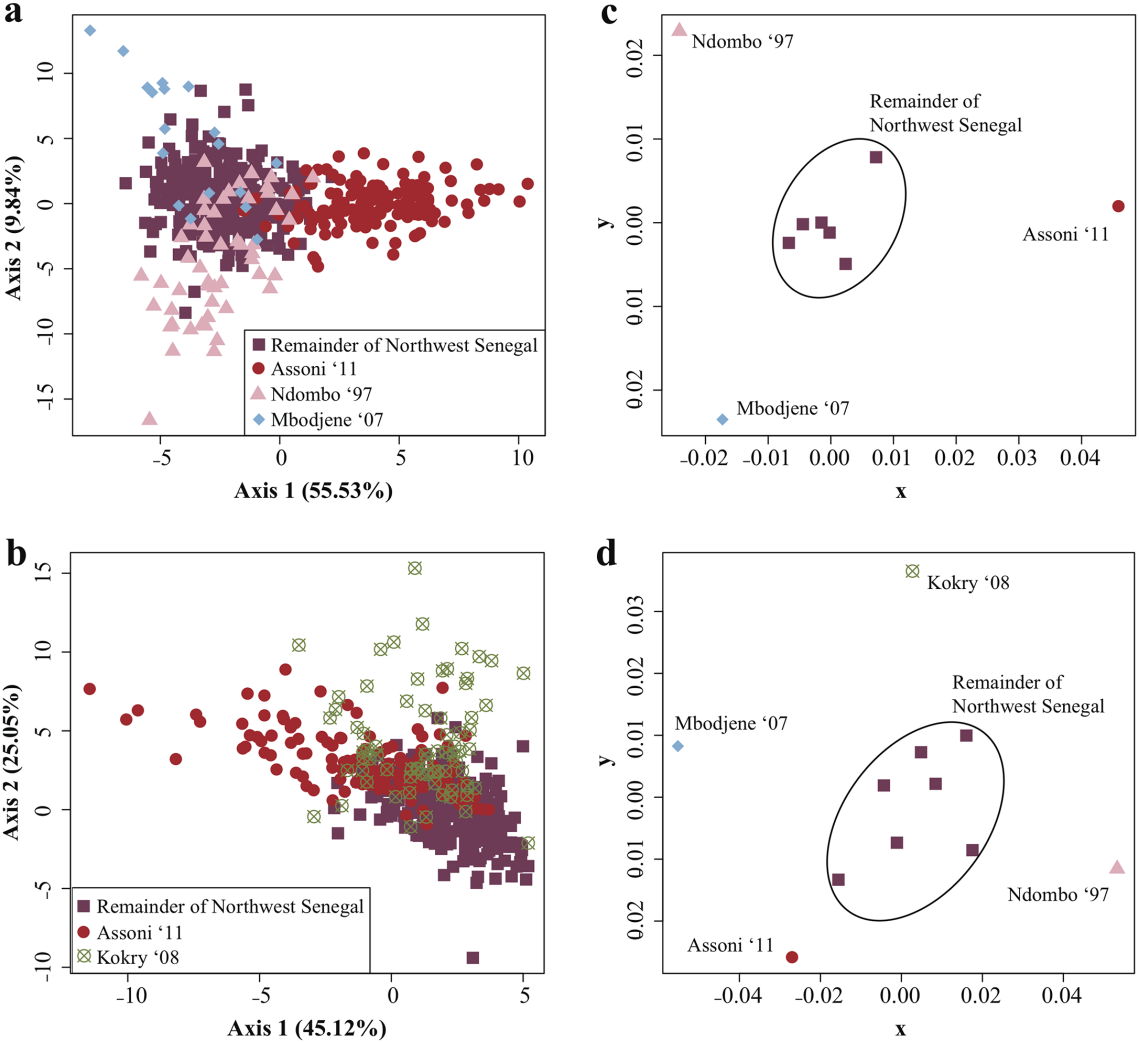
Population genetic structure of *Schistosoma mansoni* using microsatellite markers. Results of the Factorial Correspondence Analysis for datasets DMS1 (a) and DMS2 (b). Classical multidimensional scaling plots of pairwise *F*_ST_ between villages for datasets DMS1 (c) and DMS2 (d).

Factorial Correspondence analysis (FCA) for DMS2 revealed that most of the parasites from Northwest Senegal sampled in several villages from 1993 to 2007 always clustered together and differed strongly from parasites sampled in Assoni in 2011 (Southeast Senegal) and Kokry-Bozo in 2007 (Southwest Mali) (Fig 4b). For DMS1, parasites sampled in Mbodjene (2007) and Ndombo (1997) differed slightly from the remainder of Northwest Senegal, although the second axis explained only 9.84% of the total observed variation (Fig 4a).

Pairwise estimates of *F*_ST_ between regions for DMS2 were 0.064 (*p* < 0.001) between Southwest Mali and Northwest Senegal, 0.056 (*p* < 0.001) between Southwest Mali and Southeast Senegal and 0.044 (*p* < 0.001) between Northwest Senegal and Southeast Senegal. Table 4 summarizes the pairwise *F*_ST_ between villages and shows that parasite populations from Kokry-Bozo (2007), Assoni (2011), Ndombo (1997) and Mbodjene (2007) were almost always significantly differentiated from the other samples when genotypes were permuted among villages. In contrast, genetic differentiation among the other samples in Northwest Senegal was mostly low and often insignificant (Table 4). Classical multidimensional scaling (CMDS) plots based on pairwise *F*_ST_ between villages visualized this pattern (Fig 4c and 4d).

**Table 4:** Pairwise *F*_ST_ estimates between *Schistosoma mansoni* samples from Mali and Senegal. Estimates were obtained for microsatellite dataset DMS1 (below diagonal) and DMS2 (above diagonal). Note that samples with less than 10 parasites were not included to avoid biased estimation, and that samples from Richard Toll from 1993 and 1994 were pooled.

	Kokry '07	Assoni '11	Rhonne '07	Rtoll '93–94’	Rtoll '07	Ndombo '97	Theuss '06	Theuss '07	Nder '07	Ndieumeul '07	Mbodjene '07
Kokry '07		0.056**	0.059**	0.041**	0.029*	0.110**	0.067**	0.050**	0.066**	0.032**	0.065**
Assoni '11	na		0.037**	0.022**	0.014	0.083**	0.056**	0.045**	0.055**	0.027*	0.069**
Rhonne '07	na	0.039**		-0.002	0.023*	0.024**	0.016	-0.001	0.001	0.002	0.014*
Rtoll '93 –‘94	na	0.044**	-0.001		0.011	0.041**	0.017*	-0.001	0.007	-0.001	0.015
Rtoll '07	na	0.045**	0.012*	0.006		0.053**	0.038**	0.020*	0.033*	0.003	0.051**
Ndombo '97	na	0.073**	0.021**	0.025**	0.038**		0.009	0.027**	0.018**	0.012	0.066**
Theuss '06	na	na	na	na	na	na		0.012	0.016*	-0.014	0.053**
Theuss '07	na	0.049**	0.001	-0.001	0.007	0.021**	na		0.000	-0.001	0.012*
Nder '07	na	0.046**	0.002	0.007	0.020*	0.020**	na	0.003		0.003	0.012
Ndieumeul '07	na	0.028**	-0.001	-0.003	0.009	0.016*	na	0.001	0.000		0.037*
Mbodjene '07	na	0.068**	0.013*	0.023*	0.029*	0.047**	na	0.012*	0.015*	0.028**	

Kokry = short for Kokry-Bozo. Rtoll = short for Richard Toll.

* = significant for permutation of genotypes among villages at the nominal level of 0.05.

** = significant for permutation of genotypes among villages at the nominal level of 0.001 (i.e. Bonferroni corrected). na = not applicable.

## Discussion

The construction of two dams in the 1980s within the Senegal River Basin (SRB) led to a massive outbreak of intestinal schistosomiasis, a debilitating and neglected disease that had not been reported before in the region. The rate and intensity at which the epidemic expanded over Northwest Senegal was devastating: within a few years, *S*. *mansoni* was present almost everywhere in the lower valley and prevalence reached 91% in some villages [18]. Here, we investigated the level and the distribution of *S*. *mansoni* genetic diversity over a large geographic scale and wide timeframe in order to provide insight into the colonization history of this parasite in Northwest Senegal.

### Parasite genetic diversity

Our results showed that genetic diversity of *S*. *mansoni* was high, both at the mitochondrial and at the nuclear level. A total of 20 different *cox*1 haplotypes (out of 103 described across Africa) were identified in Northwest Senegal (Fig 2a). Despite the fact that mitochondrial genes are particularly prone to diversity loss after colonization events due to their haploid state and uniparental inheritance [52], a substantial level of nucleotide and haplotype diversity was detected, also for those parasite populations collected in 1993–1994 shortly after the onset of the epidemic (Table 2). This observation was confirmed by statistical parsimony analysis that showed a relatively wide range of haplotypes that were often separated by many mutations (Fig 2b). These results suggest that many *S*. *mansoni* parasites were introduced from multiple source populations. Note that, based on the commonly used 2% divergence rate for mitochondrial DNA [52], we expect none or only a few mutations in the 30 years since the beginning of the disease outbreak. At the nuclear level, levels of diversity in Northwest Senegal were relatively similar compared to Southeast Senegal and Southwest Mali (Table 3). These results suggest that there was no significant loss of genetic diversity upon introduction, and confirm that many parasites were probably introduced at the onset of the epidemic. An interesting finding in this respect is that *S*. *mansoni* genetic diversity in Richard Toll remained relatively stable since 1993 despite the many treatments during the course of the disease outbreak. Although treatment with praziquantel is expected to result in decreased population sizes and thus decreased genetic diversity [62], field-based studies revealed only a slight [63] or no decrease in diversity at all following treatment [31,64] suggesting that treatment may only have little effect on the genetic composition of natural *S*. *mansoni* populations. Note however that the actual diversity in 1993/1994 may have been larger than the one observed here, as parasites were passaged through mice, which may have induced a loss in genetic diversity.

These results highlight the success of this parasite in extending its geographic range without notable loss of genetic diversity. Maintaining genetic diversity allows the parasite to quickly adapt to a new environment or a new host, or to counteract selective pressures such as drug treatment [65,66]. Such a strong evolutionary potential could explain why *Schistosoma* parasites continue to (re-)emerge successfully into new regions [67], and why caution is warranted in any future anthropogenic environmental changes involving creation of potential new transmission sites.

### Parasite population structure

Multidimensional scaling of pairwise *F*_ST_, Factorial Correspondence Analysis and STRUCTURE analysis revealed the presence of three genetic populations in Northwest Senegal: one dominant *S*. *mansoni* population that is present in almost all villages and at all times, and two smaller populations in Ndombo 1997 and in Mbodjene 2007 (Figs 3 and 4 and Table 4). The presence of one widespread population suggests that most *S*. *mansoni* parasites originated from one and the same source population (scenario 1), or that they are the result of intraspecific hybridization (admixture) among multiple introduced parasite populations (scenario 2). Admixture among different introductions might produce recombinant genotypes that are characterized by a unique genetic profile if these genotypes were extensively shuffled, which will furthermore erode the genetic signal of the native range [68–71]. We favor the second scenario of admixture among multiple introductions because of the following reasons. (i) The presence of many divergent haplotypes at the non-recombining mitochondrial marker suggests that multiple introductions likely occurred (see above). (ii) Multiple introductions could have happened due to the substantial seasonal immigration of infected agricultural workers from neighboring regions in Senegal, Mauritania and Mali [11,12]. (iii) There is evidence of high parasite gene flow in Northwest Senegal, which tends to homogenize populations, as 19 out of 20 identified *cox*1 haplotypes were found within a single village (Table 2) and pairwise *F*_ST_ between most villages was low and insignificant (Table 4). (iv) The presence of two divergent populations in Ndombo (1997) and Mbodjene (2007) might support the hypothesis of multiple introductions. However, population bottlenecks followed by genetic drift could also produce divergent populations [72], which could apply to the sample from Ndombo that was obtained after laboratory passage [73,74]. Altogether, these findings support the hypothesis that the widespread dominant population of *S*. *mansoni* in Northwest Senegal is more likely the result of an admixture between multiple introductions (scenario 2) than the result of a single introduction (scenario 1). Unfortunately, our sampling in the native range is too restricted to reliably discriminate among both scenarios. Analyses of genetic structure indicated that the samples from Southeast Senegal and Southwest Mali represent separate genetic groups (Table 4 and Figs 3 and 4), suggesting that parasites were probably not introduced from these regions, or at least not from the villages that were sampled in these regions. Based on statistical parsimony networks it is clear that the putative source populations are within West Africa, as haplotypes sampled in Northwest Senegal clustered tightly with those from Southwest Senegal, Mali and some from Niger (Fig 2a). Additional sampling within neighboring regions is therefore necessary, preferably from regions such as Mauritania from where agricultural workers have immigrated [12] or from villages in Mali closer to the Senegalese border.

### Snail population structure

In light of the dynamic nature of a disease outbreak, it is remarkable that parasite genetic diversity remained relatively stable in space and time. *Schistosoma mansoni* genetic diversity in 1993–1994 from Richard Toll is of the same order of magnitude as 14 years later (Tables 2 and 3) and genetic differentiation between samples from different years (1993–1994 and 2007) and different villages was low and insignificant (Table 4). These results indicate that a successful parasite population, whether the result of a single introduction or of admixture between multiple introductions, must have established and spread quickly at the start of the epidemic. Such a colonization history could be linked to the colonization history of the intermediate snail host *B*. *pfeifferi*. Microsatellite analyses revealed that *B*. *pfeifferi* snail populations were genetically homogeneous in the region around Richard Toll, suggesting a rapid expansion of one or a few fecund lines at the expense of less fecund ones [75]. Experimental infection studies revealed that *B*. *pfeifferi* from Senegal showed unusual high vectorial capacities, with higher snail longevity and higher frequency of patent infections in combination with Senegalese *S*. *mansoni* (i.e. sympatric combination) than in combination with Cameroonian *S*. *mansoni* (i.e. allopatric combination) [76]. This was also evidenced from the extremely high *S*. *mansoni* prevalence (44%) in *B*. *pfeifferi* snails at the onset of the epidemic [20]. Altogether these results suggest local adaptation of *S*. *mansoni* to its intermediate snail host *B*. *pfeifferi* in Northwest Senegal. This could have led to priority effects in *S*. *mansoni*, lowering the establishment success of subsequent invasions and ensuring the temporal and spatial stability of the dominant *S*. *mansoni* population in Northwest Senegal [77,78].

An interesting finding is that *S*. *mansoni* parasites from Mbodjene (2007) in the Lampsar region were significantly differentiated from most other samples in the vicinity of Richard Toll. Likewise, the *B*. *pfeifferi* population sampled close to this locality was genetically different from the other two populations near the Lampsar River and the populations around Richard Toll [75]. This correspondence between host and parasite geographic structure further suggests that the genetic composition of the intermediate snail hosts could be an important factor determining establishment success of *S*. *mansoni* in a certain region. At the molecular level, this could comply with the hypothesis of a matching phenotype model where the interactions between parasite antigens and host immune receptors during the early stages of the infection determine the success or failure of the infection [79].

### Conclusions

This study is the first to reconstruct a recent epidemic of human intestinal schistosomiasis using mitochondrial and nuclear markers, revealing the evolutionary consequences of such a rapid colonization. The occurrence of many different haplotypes as revealed by the non-recombining mitochondrial marker indicated that multiple introductions occurred, while the recombining microsatellite markers pointed to the presence of mainly one widespread homogeneous population. We argue that admixture among multiple introductions generated a homogenous parasite population with a distinct genetic signature. The spatial and temporal stability of the established *S*. *mansoni* population suggest a swift local adaptation of the parasite to its intermediate snail host *B*. *pfeifferi* at the onset of the epidemic. Further research using samples from different localities in Senegal, Mali and Mauritania will help to pinpoint the putative source population(s) of this disease outbreak.

## Supporting Information

S1 Data This excel file contains the two microsatellite datasets that were used in this study. DMS1 can be found in excel tab 1 and contains 542 *S*. *mansoni* samples that were genotyped at nine microsatellite markers. DMS2 can be found in excel tab 2 and contains 758 *S*. *mansoni* samples that were genotyped at six microsatellite markers. Loci are coded in 6 digits (three digits per allele).
(XLSX)

## References

[1] Mack RN, Simberloff D, Mark Lonsdale W, Evans H, Clout M, Bazzaz FA. Biotic invasions: causes, epidemiology, global consequences and control Ecol Appl. Ecological Society of America 2000;10: 689–710.

[2] Kolar CS, Lodge DM. Progress in invasion biology: predicting invaders. Trends Ecol Evol. 2001;16: 199–204. 11245943 10.1016/s0169-5347(01)02101-2

[3] Sakai AK, Allendorf FW, Holt JS, Lodge DM, Molofsky J, With KA, et al The population biology of invasive species. Annu Rev Ecol Syst. Annual Reviews. 2001;32: 305–332.

[4] Suarez A V, Tsutsui ND. The evolutionary consequences of biological invasions. Mol Ecol. 2008;17: 351–360. 10.1111/j.1365-294X.2007.03456.x 18173507

[5] Torchin ME, Lafferty KD, Dobson AP, McKenzie VJ, Kuris AM. Introduced species and their missing parasites. Nature. 2003;421: 628–630. 12571595 10.1038/nature01346

[6] Mitchell CE, Power AG. Release of invasive plants from fungal and viral pathogens. Nature. 2003;421: 625–627. 12571594 10.1038/nature01317

[7] Gaither MR, Aeby G, Vignon M, Meguro Y ichiro, Rigby M, Runyon C, et al An invasive fish and the time-lagged spread of its parasite across the hawaiian archipelago. PLoS One. 2013;8. 10.1371/journal.pone.0056940 PMC3584140 23468894

[8] Novak CW, Goater TM. Introduced bullfrogs and their parasites: *Haematoloechus longiplexus* (Trematoda) exploits diverse damselfly intermediate hosts on vancouver Island. J Parasitol. 2013;99: 59–63. 10.1645/GE-3145.1 22924931

[9] Rollinson D, Simpson AJG. The biology of schistosomes From genes to latrines. London: Academic Press Ltd; 1987.

[10] Verheye WH. Impact of climate and soil-conditions on conception and implementation of irrigation schemes in the Senegal River Basin. Agric Water Manag. 1995;28: 73–94.

[11] Southgate VR. Schistosomiasis in the Senegal river basin: before and after the construction of the dams at Diama, Senegal and Manantali, Mali and future prospects. J Helminthol. 1997;71: 125–132. 9192711 10.1017/s0022149x00015790

[12] Handschumacher P, Dorsainville R, Diaw O-T, Hebrard G, Niang M, Herve JP. Constraints climatiques et amenagements hydroliques à propos de l’epidemie de bilharziose intestinale de RIchard-Toll (Sénégal) ou la modification des risques sanitaire en milieu sahelian. Risques Pathol Rythm Paroxysmes Clim. 1992; 287–295.

[13] Talla I, Kongs A, Verle P, Belot J, Sarr S, Coll AM. Outbreak of intestinal schistosomiasis in the Senegal River Basin. Ann Soc Belg Med Trop (1920). 1990;70: 173–180. 2122819

[14] Vercruysse J, Southgate VR, Rollinson D, Declercq D, Sacko M, Debont J, et al Studies on transmission and schistosome interactions in Senegal, Mali and Zambia. Trop Geogr Med. 1994;46: 220–226. 7825224

[15] Talla I, Kongs A, Verle P. Preliminary study of the prevalence of human schistosomiasis in Richard-Toll (the Senegal river basin). Trans R Soc Trop Med Hyg. 1992;86: 182 1440783 10.1016/0035-9203(92)90562-q

[16] Gryseels B, Stelma FF, Talla I, van Dam GJ, Polman K, Sow S, et al Epidemiology, immunology and chemotherapy of *Schistosoma mansoni* infections in a recently exposed community in Senegal. Trop Geogr Med. 1994;46: 209–219. 7825223

[17] Verle P, Stelma F, Desreumaux P, Dieng A, Diaw O, Kongs A, et al Preliminary study of urinary schistosomiasis in a village in the delta of the Senegal River Basin, Senegal. Trans R Soc Trop Med Hyg. 1994;88: 401–405. 7570817 10.1016/0035-9203(94)90400-6

[18] Picquet M, Ernould JC, Vercruysse J, Southgate VR, Mbaye A, Sambou B, et al The epidemiology of human schistosomiasis in the Senegal river basin. Trans R Soc Trop Med Hyg. 1996;90: 340–346. 8882173 10.1016/s0035-9203(96)90501-5

[19] Vercruysse J, Southgate VR, Rollinson D. The Epidemiology of human and animal schistosomiasis in the Senegal River Basin. Acta Trop. 1985;42: 249–259. 2865881

[20] Diaw OT, Vassiliades G, Seye M, Sarr Y. Epidemiology of intestinal schistosomiasis with *Schistosoma mansoni* in Richard-Toll (Delta of Senegal River)—Malacological Survey. Bull La Soc Pathol Exot. 1991;84: 174–183. 1914048

[21] Sow S, de Vlas SJ, Engels D, Gryseels B. Water-related disease patterns before and after the construction of the Diama dam in northern Senegal. Ann Trop Med Parasitol. 2002;96: 575–586. 12396320 10.1179/000349802125001636

[22] Stelma FF, Talla I, Sow S, Kongs A, Niang M, Polman K, et al Efficacy and side effects of praziquantel in an epidemic focus of *Schistosoma mansoni* . Am J Trop Med Hyg. 1995;53: 167–170. 7677219 10.4269/ajtmh.1995.53.167

[23] Southgate V, Tchuem Tchuente LA, Sene M, De Clercq D, Theron A, Jourdane J, et al Studies on the biology of schistosomiasis with emphasis on the Senegal river basin. Mem Inst Oswaldo Cruz. 2001;96 Suppl: 75–78. 11586429 10.1590/s0074-02762001000900010

[24] Meurs L, Mbow M, Boon N, Van den Broeck F, Vereecken K, Dièye TN, et al Micro-geographical heterogeneity in *Schistosoma mansoni* and *S*. *haematobium* infection and morbidity in a co-endemic community in Northern Senegal. PLoS Negl Trop Dis. 2013;7: e2608 10.1371/journal.pntd.0002608 24386499 PMC3873272

[25] Meurs L, Mbow M, Vereecken K, Menten J, Mboup S, Polman K. Epidemiology of mixed *Schistosoma mansoni* and *Schistosoma haematobium* infections in northern Senegal. Int J Parasitol. 2012;42: 305–311. 10.1016/j.ijpara.2012.02.002 22366733

[26] Gryseels B, Stelma F, Talla I, Polman K, Van Dam G, Sow S, et al Immuno-epidemiology of *Schistosoma mansoni* infections in a recently exposed community in Senegal. Memorias do Inst Oswaldo Cruz. 1995;90: 271–276. 10.1590/s0074-02761995000200025 8531670

[27] Polman K, Stelma FF, De Vlas SJ, Sow S, Fathers L, Le Cessie S, et al Dynamics of egg counts and circulating antigen levels in a recent *Schistosoma mansoni* focus in northern Senegal. Trop Med Int Heal. 2001;6: 538–544. 10.1046/j.1365-3156.2001.00742.x 11469948

[28] Polman K, Stelma FF, Le Cessie S, De Vlas SJ, Falcão Ferreira STM, Talla I, et al Evaluation of the patterns of *Schistosoma mansoni* infection and re-infection in Senegal, from faecal egg counts and serum concentrations of circulating anodic antigen. Ann Trop Med Parasitol. 2002;96: 679–689. 12537629 10.1179/000349802125001708

[29] Meurs L, Mbow M, Boon N, Vereecken K, Amoah AS, Labuda LA, et al Cytokine Responses to *Schistosoma mansoni* and *Schistosoma haematobium* in relation to infection in a co-endemic focus in Northern Senegal. PLoS Negl Trop Dis. 2014;8: e3080 10.1371/journal.pntd.0003080 25101661 PMC4125161

[30] Van den Broeck F, Meurs L, Raeymaekers J a M, Boon N, Dieye TN, Volckaert FAM, et al Inbreeding within human *Schistosoma mansoni*: do host-specific factors shape the genetic composition of parasite populations? Heredity. 2014;113: 32–41. 10.1038/hdy.2014.13 24619176 PMC4815646

[31] Huyse T, Van den Broeck F, Jombart T, Webster BL, Diaw O, Volckaert FAM, et al Regular treatments of praziquantel do not impact on the genetic make-up of *Schistosoma mansoni i*n Northern Senegal. Infect Genet Evol. 2013;18: 100–5. 10.1016/j.meegid.2013.05.007 23684792

[32] Kaňuch P, Berggren Å, Cassel-Lundhagen A. Genetic diversity of a successful colonizer: Isolated populations of *Metrioptera roeselii* regain variation at an unusually rapid rate. Ecol Evol. 2014;4: 1117–1126. 10.1002/ece3.1005 24772287 PMC3997326

[33] Swaegers J, Mergeay J, Therry L, Larmuseau MHD, Bonte D, Stoks R. Rapid range expansion increases genetic differentiation while causing limited reduction in genetic diversity in a damselfly. Heredity. 2013;111: 422–9. 10.1038/hdy.2013.64 23820582 PMC3806023

[34] Kolbe JJ, Glor RE, Schettino LR, Lara AC, Larson A, Losos JB. Multiple sources, admixture, and genetic variation in introduced *Anolis* lizard populations. Conserv Biol. 2007;21: 1612–1625. 10.1111/j.1523-1739.2007.00826.x 18173485

[35] Helsen K, Jacquemyn H, Hermy M, Vandepitte K, Honnay O. Rapid buildup of genetic diversity in founder populations of the gynodioecious plant species *Origanum vulgare* after semi-natural grassland restoration. PLoS One. 2013;8. 10.1371/journal.pone.0067255 PMC3686717 23840642

[36] WHO. Preventive chemotherapy in human helminthiasis. 2006; 62.

[37] Emery AM, Allan FE, Rabone ME, Rollinson D. Schistosomiasis collection at NHM (SCAN). Parasit Vectors. 2012;5: 185 22943137 10.1186/1756-3305-5-185 PMC3453491

[38] Webster BL, Webster JP, Gouvras AN, Garba A, Lamine MS, Diaw OT, et al DNA “barcoding” of *Schistosoma mansoni* across sub-Saharan Africa supports substantial within locality diversity and geographical separation of genotypes. Acta Trop. 2013;128: 250–60. 10.1016/j.actatropica.2012.08.009 22935316

[39] Katz N, Chaves A, Pellegrino J. A simple device for quantitative stool thick-smear technique in *Schistosomiasis mansoni* . Rev Inst Med Trop Sao Paulo. 1972;14: 397–400. 4675644

[40] Van den Broeck F, Geldof S, Polman K, Volckaert FAM, Huyse T. Optimal sample storage and extraction procotols for reliable multilocus genotyping of the human parasite *Schistosoma mansoni* . Infect Genet Evol. 2011;11: 1413–1418. 10.1016/j.meegid.2011.05.006 21605705

[41] Ndao B, Sy I, Talla I, Barbier D, Klotz F, Georges P. Can schistosomiasis be defeated? An example from Senegal. Med Sante Trop. 2013;23: 226. 10.1684/mst.2013.0212 24001640

[42] Gower CM, Gouvras AN, Lamberton PHL, Deol A, Shrivastava J, Mutombo PN, et al Population genetic structure of *Schistosoma mansoni* and *Schistosoma haematobium* from across six sub-Saharan African countries: Implications for epidemiology, evolution and control. Acta Trop. 2013;128: 261–274. 10.1016/j.actatropica.2012.09.014 23041540

[43] Morin PA, Manaster C, Mesnick SL, Holland R. Normalization and binning of historical and multi-source microsatellite data: overcoming the problems of allele size shift with allelogram. Mol Ecol Resour. 2009;9: 1451–1455. 10.1111/j.1755-0998.2009.02672.x 21564931

[44] Bowles J, Blair D, McManus DP. Genetic variants within the genus *Echinococcus* identified by mitochondrial DNA sequencing. Mol Biochem Parasitol. 1992;54: 165–174. 1435857 10.1016/0166-6851(92)90109-w

[45] Lockyer AE, Olson PD, Ostergaard P, Rollinson D, Johnston DA, Attwood SW, et al The phylogeny of the Schistosomatidae based on three genes with emphasis on the interrelationships of *Schistosoma* Weinland, 1858. Parasitology. 2003;126: 203–224. 12666879 10.1017/s0031182002002792

[46] Durand P, Sire C, Theron A. Isolation of microsatellite markers in the digenetic trematode *Schistosoma mansoni* from Guadeloupe island. Mol Ecol. 2000;9: 997–998. 10886664 10.1046/j.1365-294x.2000.00939-4.x

[47] Blair L, Webster JP, Barker GC. Isolation and characterization of polymorphic microsatellite markers in *Schistosoma mansoni* from Africa. Mol Ecol Notes. 2001;1: 93–95.

[48] Curtis J, Sorensen RE, Page LK, Minchella DJ. Microsatellite loci in the human blood fluke *Schistosoma mansoni* and their utility for other schistosome species. Mol Ecol Notes. 2001;1: 143–145.

[49] Edgar RC, Edgar RC. MUSCLE: multiple sequence alignment with high accuracy and high throughput. Nucleic Acids Res. 2004;32: 1792–7. 15034147 10.1093/nar/gkh340 PMC390337

[50] Librado P, Rozas J. DnaSP v5: a software for comprehensive analysis of DNA polymorphism data. Bioinformatics. 2009;25: 1451–1452. 10.1093/bioinformatics/btp187 19346325

[51] Nei M. Molecular Evolutionary Genetics. New York: Columbia University Press; 1987.

[52] Avise JC. Molecular Markers, Natural History, and Evolution The Auk. 2004.

[53] Templeton AR, Crandall KA, Sing CF. A cladistic analysis of phenotypic associations with haplotypes inferred from restriction endonuclease mapping and DNA sequence data. III. Cladogram estimation. Genetics. 1992;132: 619–633. 1385266 10.1093/genetics/132.2.619 PMC1205162

[54] Paradis E. pegas: an R package for population genetics with an integrated-modular approach. Bioinformatics. 2010;26: 419–420. 10.1093/bioinformatics/btp696 20080509

[55] R Development Core Team R. R: A language and environment for statistical computing. R Foundation for Statistical Computing 2013 p. 409.

[56] Belkhir K, Borsa P, Chikhi L, Raufaste N, Bonhomme F. GENETIX 4.05, logiciel sous Windows TM pour la génétique des populations. Montpellier (France). Laboratoire Génome, Populations, Interactions, CNRS UMR 5000, Université de Montpellier II.

[57] Goudet J. HIERFSTAT, a package for R to compute and test hierarchical *F*-statistics. Mol Ecol Notes. 2005;5: 184–186.

[58] Weir BS, Cockerham CC. Estimating *F*-Statistics for the Analysis of Population-Structure. Evolution (N Y). 1984;38: 1358–1370. 10.1111/j.1558-5646.1984.tb05657.x 28563791

[59] Pritchard JK, Stephens M, Donnelly P. Inference of population structure using multilocus genotype data. Genetics. 2000;155: 945–959. 10835412 10.1093/genetics/155.2.945 PMC1461096

[60] Hubisz MJ, Falush D, Stephens M, Pritchard JK. Inferring weak population structure with the assistance of sample group information. Mol Ecol Resour. 2009;9: 1322–1332. 10.1111/j.1755-0998.2009.02591.x 21564903 PMC3518025

[61] Besnier F, Glover KA. ParallelStructure: A R package to distribute parallel runs of the population genetics program STRUCTURE on multi-core computers. PLoS One. 2013;8. 10.1371/journal.pone.0070651 PMC3726640 23923012

[62] Coeli R, Baba EH, Araujo N, Coelho PMZ, Oliveira G. Praziquantel treatment decreases *Schistosoma mansoni* genetic diversity in experimental infections. PLoS Negl Trop Dis. 2013;7: e2596 10.1371/journal.pntd.0002596 24367712 PMC3868512

[63] Norton AJ, Gower CM, Lamberton PH, Webster BL, Lwambo NJ, Blair L, et al Genetic consequences of mass human chemotherapy for *Schistosoma mansoni*: population structure pre- and post-praziquantel treatment in Tanzania. Am J Trop Med Hyg. 2010;83: 951–957. 10.4269/ajtmh.2010.10-0283 20889898 PMC2946775

[64] Blanton RE, Blank WA, Costa JM, Carmo TM, Reis EA, Silva LK, et al *Schistosoma mansoni* population structure and persistence after praziquantel treatment in two villages of Bahia, Brazil. Int J Parasitol. 2011;41: 1093–1099. 10.1016/j.ijpara.2011.06.002 21784077 PMC3155667

[65] McDonald BA, Linde C. Pathogen population genetics, evolutionary potential, and durable resistance. Annu Rev Phytopathol. 2002;40: 349–379. 12147764 10.1146/annurev.phyto.40.120501.101443

[66] Barrett LG, Thrall PH, Burdon JJ, Linde CC. Life history determines genetic structure and evolutionary potential of host-parasite interactions. Trends Ecol Evol. 2008;23: 678–685. 10.1016/j.tree.2008.06.017 18947899 PMC2653456

[67] Chitsulo L, Engels D, Montresor A, Savioli L. The global status of schistosomiasis and its control. Acta Trop. 2000;77: 41–51. 10996119 10.1016/s0001-706x(00)00122-4 PMC5633072

[68] Ellstrand NC, Schierenbeck KA. Hybridization as a stimulus for the evolution of invasiveness in plants? Euphytica. 2006;148: 35–46. 10.1073/pnas.97.13.7043 PMC34382 10860969

[69] Rosenthal DM, Ramakrishna AP, Cruzan MB. Evidence for multiple sources of invasion and intraspecific hybridization in *Brachypodium sylvaticum* (Hudson) Beauverin North America. Mol Ecol. 2008;17: 4657–4669. 10.1111/j.1365-294X.2008.03844.x 18627455

[70] Keller SR, Taylor DR. Genomic admixture increases fitness during a biological invasion. J Evol Biol. 2010;23: 1720–1731. 10.1111/j.1420-9101.2010.02037.x 20626546

[71] Verhoeven KJF, Macel M, Wolfe LM, Biere A. Population admixture, biological invasions and the balance between local adaptation and inbreeding depression. Proc Biol Sci. 2011;278: 2–8. 10.1098/rspb.2010.1272 20685700 PMC2992731

[72] Hartl D, Clark A. Principles of Population genetics. 4th ed Sunderland, Massachusetts, USA: Sinauer Associates, Inc. Publishers; 2007.

[73] Bech N, Beltran S, Portela J, Rognon A, Allienne JF, Boissier J, et al Follow-up of the genetic diversity and snail infectivity of a *Schistosoma mansoni* strain from field to laboratory. Infect Genet Evol. 2010;10: 1039–1045. 10.1016/j.meegid.2010.06.012 20601175

[74] Blank WA, Test MR, Liu SF, Lewis FA, Blanton RE. Long-term genetic stability and population dynamics of laboratory strains of *Schistosoma mansoni* . J Parasitol. 2010;96: 900–907. 10.1645/GE-2463.1 20950096 PMC3881424

[75] Campbell G, Noble LR, Rollinson D, Southgate VR, Webster JP, Jones CS. Low genetic diversity in a snail intermediate host (*Biomphalaria pfeifferi* Krass, 1848) and schistosomiasis transmission in the Senegal River Basin. Mol Ecol. 2010;19: 241–256. 10.1111/j.1365-294X.2009.04463.x 20025653

[76] Southgate VR, Tchuente LAT, Theron A, Jourdane J, Ly A, Moncrieff CB, et al Compatibility of *Schistosoma mansoni* Cameroon and *Biomphalaria pfeifferi* Senegal. Parasitology. 2000;121: 501–505. 11128801 10.1017/s0031182099006708

[77] De Meester L, Gómez A, Okamura B, Schwenk K. The Monopolization Hypothesis and the dispersal-gene flow paradox in aquatic organisms. Acta Oecologica. 2002 pp. 121–135.

[78] Orsini L, Vanoverbeke J, Swillen I, Mergeay J, De Meester L. Drivers of population genetic differentiation in the wild: Isolation by dispersal limitation, isolation by adaptation and isolation by colonization. Molecular Ecology. 2013 pp. 5983–5999. 10.1111/mec.12561 24128305

[79] Mitta G, Adema CM, Gourbal B, Loker ES, Theron A. Compatibility polymorphism in snail/schistosome interactions: From field to theory to molecular mechanisms. Dev Comp Immunol. 2012;37: 1–8. 10.1016/j.dci.2011.09.002 21945832 PMC3645982

