# Correction: Reconstructing Colonization Dynamics of the Human Parasite *Schistosoma mansoni* following Anthropogenic Environmental Changes in Northwest Senegal

**DOI:** 10.1371/journal.pntd.0004090

**Published:** 2015-09-25

**Authors:** Frederik Van den Broeck, Gregory E. Maes, Maarten H. D. Larmuseau, David Rollinson, Ibrahima Sy, Djibril Faye, Filip A. M. Volckaert, Katja Polman, Tine Huyse

There is an omission in the Acknowledgments section. Please see the corrected Acknowledgments here: We thank Prof. J. Webster, together with Drs. Polydor Ngoy Mutombo, Moussa Sacko and Charlotte Gower, for access to the raw microsatellite data of Kokry-Bozo (Mali), and the Schistosomiasis Collection team at the Natural History Museum in London (SCAN) for providing *S*. *mansoni* worms. We are grateful to all villagers in Senegal for their cooperation and thank the Senegalese field team (M Diop, A Fall, N Sy, M Wade, A Yague) for their dedicated help with the fieldwork.

## References

[1] Van den Broeck F, Maes GE, Larmuseau MHD, Rollinson D, Sy I, Faye D, et al (2015) Reconstructing Colonization Dynamics of the Human Parasite *Schistosoma mansoni* following Anthropogenic Environmental Changes in Northwest Senegal. PLoS Negl Trop Dis 9(8): e0003998 doi:10.1371/journal.pntd.0003998 26275049 10.1371/journal.pntd.0003998 PMC4537236

